# Transcript Profiling Identifies Early Response Genes against FMDV Infection in PK-15 Cells

**DOI:** 10.3390/v10070364

**Published:** 2018-07-11

**Authors:** Tianliang Zhang, Haotai Chen, Linlin Qi, Jie Zhang, Run Wu, Yongguang Zhang, Yuefeng Sun

**Affiliations:** 1 State Key Laboratory of Veterinary Etiological Biology, OIE/National Foot and Mouth Disease Reference Laboratory, Key Laboratory of Animal Virology of Ministry of Agriculture, Lanzhou Veterinary Research Institute, Chinese Academy of Agricultural Sciences, Lanzhou 730046, China; zhangtl7038@163.com (T.Z.); chenhaotai@caas.cn (H.C.); qilinlin@caas.cn (L.Q.); zhangjie03@caas.cn (J.Z.); 2 College of Veterinary Medicine, Gansu Agricultural University, Lanzhou 730070, China; wurun@gsau.edu.cn; 3 Jiangsu Co-Innovation Center for Prevention and Control of Important Animal Infectious Diseases and Zoonoses, Yangzhou 225009, China

**Keywords:** FMDV, PK-15 cells, RNA-seq, RT-qPCR, inflammation

## Abstract

Foot-and-mouth disease (FMD) is a highly contagious disease that results in enormous economic loses worldwide. Although the protection provided by vaccination is limited during early infection, it is recognized as the best method to prevent FMD outbreaks. Furthermore, the mechanism of host early responses against foot-and-mouth disease virus (FMDV) infection remains unclear. In our study, a pig kidney cell line (PK-15) was used as a cell model to reveal the mechanism of early pig responses to FMDV infection. Four non-treated control and four FMDV-treated PK-15 cells were sequenced with RNA-seq technology, and the differentially expressed genes (DEGs) were analyzed. The results showed that 1212 DEGs were in the FMDV-infected PK-15 cells, including 914 up-regulated and 298 down-regulated genes. Kyoto Encyclopedia of Genes and Genomes (KEGG) pathways were significantly enriched in the tumor necrosis factor (TNF), cytokine-cytokine receptor interaction, NOD-like receptor, toll-like receptor, NF-κB, and the chemokine signaling pathways. To verify the results of the DEGs, 30 immune-related DEGs (19 up-regulated and 11 down-regulated) were selected for Quantitative Reverse Transcriptase polymerase chain reaction (RT-qPCR) verification. The results showed that RT-qPCR-measured genes exhibited a similar pattern as the RNA-seq analyses. Based on bioinformatics analysis, during FMDV early infection, we found that a series of cytokines, such as interleukins (IL6), chemokines (CXCL2, CCL20 and CCL4), and transcription factors (ZFP36, FOS, NFKBIA, ZBTB3, ZNF503, ZNF283, dymeclin (DYM), and orthodenticle homeobox 1 (OTX1)) were involved in the battle between FMDV and the host. Combined with their features and functions, we propose inflammation as the main early mechanism by which the host responds to FMDV infection. These data provide an additional panel of candidate genes for deciphering the mechanisms of a host’s early response against FMDV infection.

## 1. Introduction

Foot-and-mouth disease (FMD) is a highly contagious disease of cloven-hoofed animals [1] which is endemic in many regions of the world, causing enormous economic losses due to reduced productivity and trade restrictions [2]. According to the World Organization for Animal Health, FMD is a notifiable disease of ruminants. Vaccination is recognized as the best method for preventing FMD outbreaks [3]. However, the use of FMD vaccines to protect against early infection is limited [4]. Additionally, the mechanism of the host’s early responses against foot-and-mouth disease virus (FMDV) infection remains unclear. Therefore, a novel strategy is required to control early-stage FMDV infection.

Foot-and-mouth disease virus (FMDV) belongs to the genus *Aphthovirus* in the family *Picornaviridae* [5] and its infection can have multiple effects on the host. During early FMDV infection, the innate immune system of the host is activated to establish an antiviral state [6]. The innate immune response provides the first line of defense, which is crucial for preventing infection [7]. To detect and respond to vastly different groups of pathogens, the innate immune system uses several recognition systems that rely on sensing common structural and functional features of pathogens [8]. The pathogen recognition receptors (PRRs) recognize conserved microbial structures, collectively termed pathogen-associated molecular patterns (PAMPs) and report to the host [9]. Most PRRs can be classified into one of five families based on protein domain homology. These five families consist of the Toll-like receptors (TLRs), C-type lectin receptors (CLRs), nucleotide binding domain, leucine-rich repeat (LRR)-containing (or NOD-like) receptors (NLRs), RIG-I like receptors (RLRs), and the AIM2-like receptors (ALRs) [10]. Using lentivirus-driven RNA interference, Husser et al. [9] reported that FMDV was recognized by MDA5 in PK-15 cells. Zhu et al. [11] observed that the expression of RIG-I inhibited FMDV replication occurred in a dose-dependent manner whereas an enhancement was observed in RIG-I siRNA PK-15 cells. MDA 5 and RIG-1 were classified into the RLRs family [12]. In addition, Ping Du [13] reported that heparin sulfate may be the main receptor for CHA/99 strain attachment-susceptible cells. Integrins are used by many viruses as a receptor for cell entry, but, as for FMDV, the flexibility of the integrin binding portions of the virus has made it difficult to visualize the interaction [14]. The cells that sense infections produce one set of cytokines, which then induce lymphocytes to produce another set of cytokines, which in turn activate effector responses [8]. The main components of the innate immune system include the complement system, plasma proteins, professional phagocytes, and natural killer cells [15]. A major component of a PRR-induced innate immune response is transcriptional, which leads to the production of proinflammatory cytokines and interferons (IFN) [16]. Cytokines are small, non-enzymatic glycoproteins that play an essential role in the innate immune system as a critical component of the protein system. PRR activation also initiates non-transcriptional responses such as the induction of phagocytosis, autophagy, cell death, and cytokine processing [16]. In many instances, individual cytokines have multiple biological activities; different cytokines can also have the same activity, which provides functional redundancy in regulating inflammatory and immune systems [17].

Pigs (*Sus scrofa*) are a major natural host of FMDV and have been used as a challenge model in many studies [18]. In this study, pig kidney cell line (PK-15) was used as a cell model to investigate the early immune responses against FMDV infection. In this project, to deeply understand the mechanism of the host’s early response against FMDV infection at the mRNA level, we performed transcriptome analysis during a FMDV infection to investigate the host’s gene expression pattern. Based on the high-throughput sequencing of mRNA (RNA-seq) and RT-qPCR validation, we analyzed the differentially expressed genes (DEGs) between FMDV-treated (FMDV) and non-treated control (NC) cells. Our data clearly showed that FMDV infection induced dramatic changes in the host’s gene expression pattern after being infected with FMDV for one hour.

## 2. Materials and Methods

### 2.1. Cells and Virus

Porcine kidney (PK-15) cells with an 18th passage number were preserved in our own laboratory and cultured in Dulbecco’s Modified Eagle Medium (DMEM) (Gibco, Grand Island, NY, USA) (All abbreviations’ information shown in Supplementary Table S1), supplemented with 10% fetal bovine serum (FBS) (AusGeneX, Brisbane, Australia). Cells were incubated at 37 °C with 5% CO_2_. Foot-and-Mouth Disease Virus Type O/BY/2010 (FMDV O/BY2010) was cultured and propagated in BHK-21 cells. The 50% tissue culture infective doses (TCID_50_) were calculated according to the well-number with cytopathic effect (CPE) using the Reed and Muench method after growing at 37 °C for 48 h. All virus-related experiments were conducted in the ABSL-3 Laboratory of Lanzhou Veterinary Research Institute according to the protocol provided by the biosafety management committee.

### 2.2. Cell Treatment and RNA Extraction

Four 60-mm dishes, seeded with monolayer of PK-15 cells growing in a logarithmic phase, were incubated for 1 h at 37 °C with FMDV at the multiplicity of infection (MOI) of 0.1 to allow virus enter cells (Supplementary Figures S1 and S2). As a control, another four 60-mm dishes, seeded with PK-15 cells, were cultured in same volume of DMEM (FBS-free) for 1 h at 37 °C. After 1 h, the supernatant was removed and washed three times with pre-chilled PBS. Then 1 mL TRIzol Reagent (Invitrogen, Carlsbad, CA, USA) was added to each dish and cells were collected, and total RNA was extracted according to the manufacturer’s protocol (Tiangen Biotech, Beijing, China).

### 2.3. Sample Preparation and Quality Control (QC)

Eight samples (four samples were cultured in FBS-free DMEM as the NC and four were infected with FMDV O/BY/2010 as the FMDV) were analyzed at the transcriptome level using RNA-Seq at CapitalBio Technology Inc. (Beijing, China) using Illumina High-throughput sequencing technology. The detailed protocol was as follows. Total RNA was prepared as a starting sample for library construction of mRNA sequencing. The amount of RNA for analysis was 1 μg, which was accurately quantified using the QUBIT RNA ASSAY KIT (Invitrogen, Carlsbad, CA, USA). The quality of each sample was controlled using the Agilent 2100 BioAnalyzer (RNA 6000 Pico chip, Agilent, Cat NO. 5067–1524,Santa Clara, CA, USA), including RNA Integrity Number (RIN) ≥ 7; 28S:18S ratio ≥ 1.5.

### 2.4. Library Construction and Sequencing

Oligo (dT) magnetic beads were used to selectively bind mRNA with the polyA tail, then the purified mRNA was fragmented and reverse transcribed to double-strand cDNA (ds cDNA) and purified with AMPure XP Beads (QUBIT DNA BR ASSAY KIT, Invitrogen, Carlsbad, CA, USA, Cat No. Q32855). The ds cDNAs were treated with End Repair/dA-tail by using the NEBNext Ultra RNA Library Prep Kit for Illumina (NEB, Ipswich, MA, USA, Cat No. E7530S). We then proceeded immediately to Adaptor Ligation with NEBNext Multiplex Oligos for Illumina (NEB, Ipswich, MA, USA, Cat No. E7335S). Universal and index PCR primers were used to amplify the ligation product. PCR products were accurately quantified using the QUBIT DNA HS ASSAY KIT (Invitrogen, Carlsbad, CA, USA). Then the Agilent 2100 Bioanalyzer chip (Agilent, Facility No. J06-02, Santa Clara, CA, USA) was used to determine whether the fragment size of the PCR products met the requirements of subsequent sequencing. The RT-qPCR was performed to absolutely quantify the constructed library by using the KAPA SYBR^®^ FAST qPCR master Mix (Cat No. KK4602, Kapa Biosystems, Wilmington, MA, USA) and DNA Quantification Standards and Primer Premix Kit (Cat No. KK4808, Kapa Biosystems, Wilmington, MA, USA). Based on the prepared library, sequence data were generated on an Illumina HiSeq 2500 (Illumina Inc., San Diego, CA, USA), using HiSeq Rapid SBS Kit v2 (Illumina, Inc. San Diego, CA, USA).

### 2.5. Sequencing Data Analysis

Raw sequencing reads were analyzed with FastQC and NGSQC for quality control and filtering. Clean reads were screened from raw reads under the following criteria: If the N content in the read exceeded 5%, the entire pair of reads was removed; if the low-quality base in the read (the mass value was not higher than 20 bases) exceeded 30%, the entire pair of reads was removed; and if the read contained the linker sequence, the entire pair of reads was removed. HISAT was used for sequence matching (also called sequence alignment), which reflected the sample data’s specific location in the reference genome (ENSEMBL: release-83/Sscrofa10; Link of Reference genome file: ftp://ftp.ensembl.org/pub/release-83/fasta/sus_scrofa/dna/ and link of reference gene annotation file: ftp://ftp.ensembl.org/pub/release-83/gff3/sus_scrofa/). Then, by using Picard software, we obtained the reads’ matching area in the reference genome. Cufflinks was used to assemble the short reads into contigs for each sample. Cuffmerge was used to merge the assemble results of all samples and obtain a total annotation file of the transcriptomes. Cuffdiff was used to analyze the differentially expressed genes (DEGs) between samples. After independent statistical hypothesis testing was performed in the analysis of the DEGs, the FDR method was used to obtain the corrected *p*-value, where the smaller the *p*-value, the more significant the differences in gene expression.

### 2.6. Functional Annotation and Differential Expression Analysis of Unigenes

Fragments per kilo bases per million fragments method (FPKM) was used to evaluate the expression level of Unigene and the fold-change between different samples. Blastx and Blast2GO were used to compare the Unigene sequence with the gene ontology (GO) database (http://www.geneontology.org/). The protein with the highest sequence similarity to Unigene was obtained to annotate the Unigene’s protein functional information. Then WEGO was used to count the GO functional classification of all Unigenes to understand the characteristics of the gene functional distribution. GO can be divided into three groups: cellular component, molecular function, and biological process. Pathway enrichment analysis of DEGs was performed by comparing the Unigenes to the KEGG database. The KEGG pathway includes seven categories: Metabolism, genetic information processing, environmental information processing, cellular processes, organismal systems, human diseases, and drug development. For detailed information, please refer to the KEGG official website (http://www.genome.jp/kegg/pathway.html). The genes analyzed in this project were annotated by combining multiple reference databases including ENSEMBL, NCBI, Uniprot, GO, and KEGG with reference genes used in the species.

### 2.7. RT-QPCR Validation of DEGs

The extraction of total RNA and the synthesis of first-strand cDNA were previously described. cDNA diluted 20-fold was used as a template and RT-qPCR was performed on a CFX96 Real Time PCR System (Bio-Rad, Hercules, CA, USA). A reaction volume of 20 μL was used containing 10 μL of SYBR Green qPCR Master Mix (Takara, Shiga, Japan), 2 μL of diluted cDNA, and 4 μL of each primer (1 μM) (Supplementary Table S2). The RT-qPCR cycling program was performed at 95 °C for 2 min, followed by 40 cycles at 95 °C for 10 s, 61 °C for 30 s, and 72 °C for 30 s. Then a melting curve was constructed from 55 to 98 °C. The relative expression of each gene comparing the FMDV-infected PK-15 cells to uninfected cells was normalized using the reference gene (β-actin) using the 2^−ΔΔCT^ calculation method: ΔΔCT = (CT_Target_ − CT_β-actin_)_FMDV_ − (CT_Target_ − CT_β-actin_)_NC_. Differences were analyzed and graphed using the GraphPad Prism 5 software with the Student’s *t*-test. *p* < 0.05 was recognized as statistically significant.

## 3. Results

### 3.1. Quality Control of Sequencing Data

To investigate the early response of the pig immune system to FMDV infection at the transcriptional level, we sequenced eight samples by using RNA sequencing technology. The sequencing generated a total of 672,190,942 raw sequencing reads and 635,330,654 clean reads (94.52% of the total) after filtering out the low-quality reads. The average value of Q20 and Q30 were 97.39375 and 93.00875, respectively (Table 1), meeting the experimental requirements. The resulting sequence reads were aligned with the reference genome and the average proportion of mapped reads was 77.44% (Table 2). Among them, the average proportion of uniquely mapped reads and multiple mapped reads were 89.38 and 10.62%, respectively. The above results suggest that the quality of sequencing was satisfactory for further analysis.

### 3.2. Assembling Transcripts

**Figure 1:**
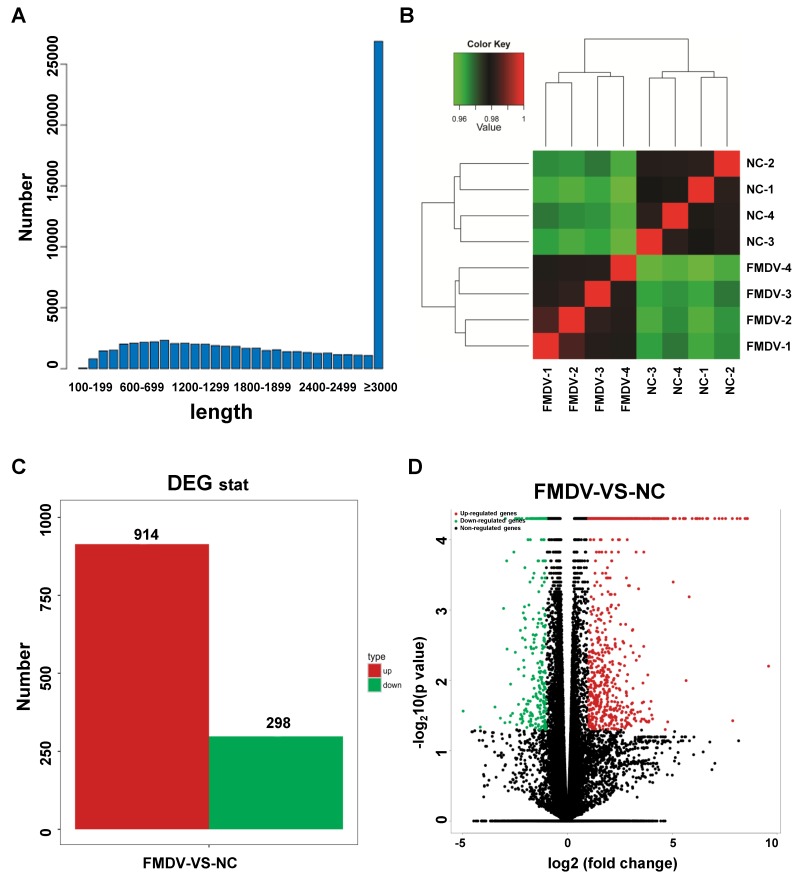
Quality control and statistics of differentially expressed genes (DEGs). (**A**) Length distribution of the assembled transcript, where the *x*-axis represents the length distribution and the *y*-axis indicates the number of transcripts at each length. More than 25,000 transcripts had a length equal to or greater than 3000, which was the largest proportion. (**B**) Sample correlation heat map. The green to red color key indicates the Pearson correlation coefficient (R) value, representing lower to higher *r*-values, respectively, suggesting the sample repeatability in the same group and also reflecting the differences between the two groups. (**C**) Number of differential genes between foot-and-mouth disease virus (FMDV)-treated and non-treated groups. Red represents up-regulated genes and green represents down-regulated genes. The *y*-axis indicates the number of genes. (**D**) Volcano plots of the distribution of DEGs. The *x*-axis indicates the fold changes of DEGs; the greater the absolute *x*-value, the greater the fold change. The *y*-axis represents the significance of the DEGs; the greater the *y*-value, the smaller the *p*-value. Red dots represent up-regulated genes, green dots represent down-regulated genes, and black dots represent not significantly regulated genes.

After quality control and alignment, the transcripts of each sample were assembled. As shown in Table 3, in accordance with the alignment results, each sample was separately assembled. The results include gene number, transcript number, and N50 length without intron. Importantly, the average of the maximum transcript length (bp), minimum transcript length (bp), average transcript length (bp), and N50 length were 23,977, 144, 2002, and 2847, respectively. The total number of transcripts with a length greater than or equal to 3000 was more than 25,000 (Figure 1A). Results of all samples were combined to generate a single annotation file with the corresponding reference genome annotation information. After screening and deleting the transcripts with total exons below 100 bp, we obtained a final GTF transcripts-annotated document.

### 3.3. Detection of Sample Repeatability

Biological replicates are important for RNA-seq experiments. Sample correlation analysis can assist in screening abnormal samples between biological repeated samples. Therefore, to ensure highly reliable repeatability, the Pearson correlation coefficient (R) was calculated using SPSS 19.0 software to quantify the degree of correlation between repeated samples. The absolute value of R was less than or equal to 1 (|R| ≤ 1). As shown in Figure 1B and Supplementary Table S4, in our experiments, the R value of each sample was greater than 0.95 and the lowest value was 0.95656192, demonstrating high reliability. These data showed that our results are reproducible and available for subsequent analysis.

### 3.4. Analysis of Differentially Expressed Genes (DEGs)

To investigate the gene expression changes in PK-15 after FMDV infection, all expressed genes (up-, down-, and non- regulated genes) in each FMDV-vs.-NC pair were counted with an independent statistical hypothesis test, which yielded differential test *p*-values. *q*-Values were obtained through the corrected *p*-values using the FDR method. The lower the *p*- or *q*-value, the more significant the difference in gene expression. As shown in Figure 1C, compared with non-treated control samples, after being treated with FMDV for 1 h, a total of 914 significantly up-regulated genes and 298 down-regulated genes were screened under the criteria of a minimum of a two-fold-change and a *p*-value < 0.05. The volcano plots of all expressed genes, based on the results of each FMDV-NC pair, are shown in Figure 1D. These data suggest that FMDV infection induced dramatic gene expression changes in PK-15 cells, even in a short time period.

### 3.5. GO Annotation of DEGs

**Figure 2:**
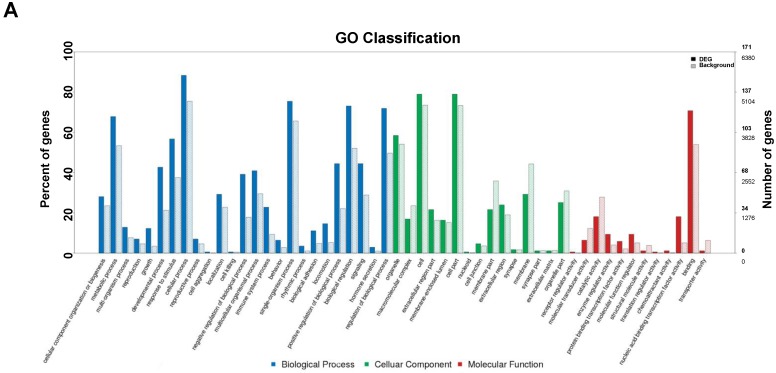

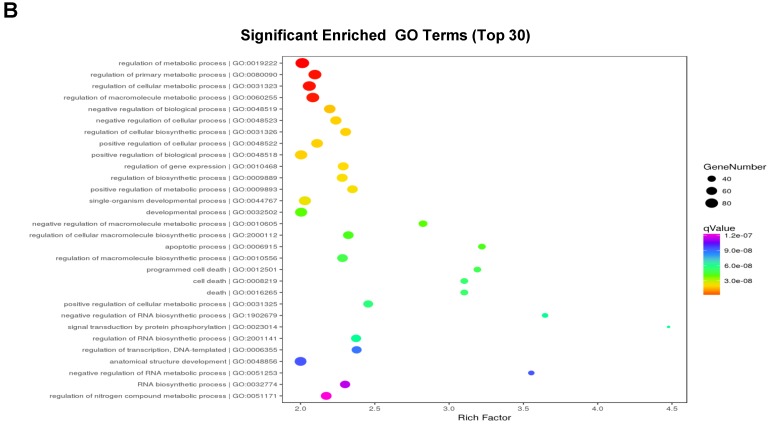
Gene ontology (GO) functional classification and enrichment analysis of DEGs. (**A**) GO functional classification of the DEGs. The *x*-axis indicates the functional description of the GO classifications, the left *y*-axis represents the proportion of the number of genes annotated to the GO database in the number of background genes, and the right *y*-axis represents the number of genes. The solid line represents the number of DEGs and the dotted line represents the total number of genes expressed in the background. All GO terms are grouped into three major categories: Blue is for biological processes, green is for cellular components, and red is for molecular function. A total of 2270 DEGs were classified to the unique gene matches, including 1375 DEGs (60.6%) to biological processes, 651 DEGs (28.7%) to cellular components, and 244 DEGs (10.7%) to molecular function. (**B**) Top 30 GO enrichment results of DEGs. The *x*-axis indicates –log (corrected *p*-value). A greater *x*-value represents a smaller corrected *p*-value, suggesting the enrichment is more significant. The *y*-axis represents the function descriptions of the GO terms. The regulation of metabolic processes is the most significant enriched GO term, followed by regulation of primary metabolic processes, regulation of cellular metabolic processes, and so forth.

To investigate the effect of FMDV infection on the host’s biological processes, the DEGs were used to perform GO annotation and enrichment analysis. The GO annotations of the DEGs were involved in three major categories: Biological processes, cellular components, and molecular function, which were divided into 52 smaller classes (Figure 2A). A total of 2270 DEGs were assigned to the unique gene matches, including 1375 DEGs (60.6%) were classified into the biological processes, which were concentrated in cellular component organization, biogenesis, metabolic processes, and multi-organism processes. A total of 651 DEGs (28.7%) were classified into the cellular component, which were significantly concentrated in organelles, macromolecular complexes, and cells. An additional 244 DEGs (10.7%) were classified into molecular function, which were significantly concentrated in receptor regulator activity, molecular transducer activity, and catalytic activity. GO enrichment analysis showed that a total of 247 significantly enriched GO terms occurred in response to FMDV infection, with a Benjamini-Hochberg (BH)-corrected *p*-value less than 0.001. Among them, the top 30 terms were mainly involved in the regulation of metabolic process (GO:0019222, GO:0080090, GO:0031323, and GO:0060255), regulation of biological process (GO:0048519 and GO:0048518), regulation of cellular processes (GO:0050794, GO:0048522, and GO:0048523), regulation of gene expression (GO:0010468), and nucleic acid binding transcription factor activity (GO:0001071) (Figure 2B). These processes mainly responded to early-stage FMDV infection.

### 3.6. KEGG Pathway Enrichment Analysis of DEGs

**Figure 3:**
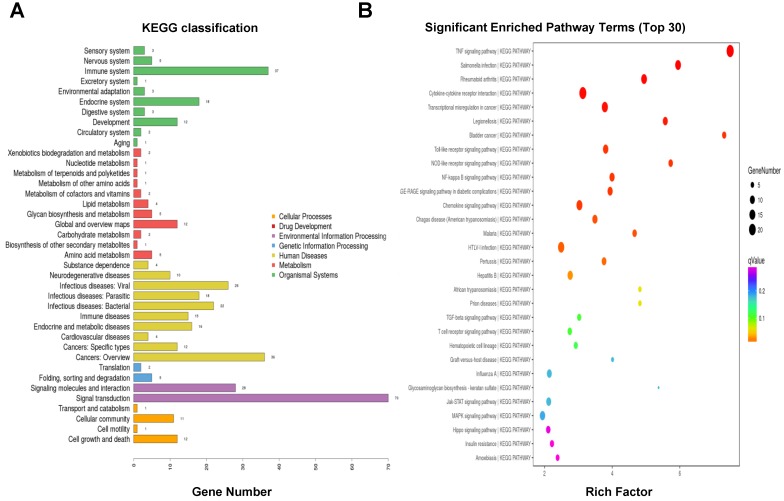
Kyoto Encyclopedia of Genes and Genomes (KEGG) classifications and pathway enrichment analysis of DEGs. (**A**) KEGG classifications of DEGs. The *x*-axis indicates the number of DEGs and the *y*-axis represents KEGG terms. All KEGG terms are grouped into seven categories: dark yellow is for cellular processes, red is for drug development, purple is for environmental information processing, light blue is for genetic information processing, light yellow is for human diseases, light red is for metabolism, and green is for organismal systems. (**B)** Top 30 pathway enrichment results for the DEGs. The *x*-axis indicates the –log (corrected *p*-value). The greater the *x*-value, the smaller the corrected *p*-value, suggesting the enrichment is more significant. The *y*-axis represents the function descriptions of the enriched pathways. The TNF signaling pathway is the most significant enriched pathway, followed by salmonella infection, rheumatoid arthritis, cytokine-cytokine receptor interaction, NOD-like receptor signaling pathway, toll-like receptor signaling pathway, NF-kappa B signaling pathway, and the chemokine signaling pathway.

To determine the associations between DEGs related to the early host responses against FMDV infection, we performed KEGG annotation and pathway enrichment analysis based on the KEGG database (http://www.genome.jp/kegg/pathway.html). As shown in Figure 3A, KEGG classifications showed the DEGs were mainly involved in signal transduction (70 genes), the immune system (37 genes), cancer (36 genes), signaling molecules and interactions (28 genes), viral infectious diseases (26 genes), bacterial infectious diseases (22 genes), the endocrine system (18 genes), parasitic infectious diseases (18 genes), endocrine and metabolic diseases (16 genes), as well as immune diseases (15 genes). The most significant enrichment pathways were the TNF signaling pathway (ssc04668), cytokine-cytokine receptor interaction (ssc04060), NOD-like receptor signaling pathway (ssc04621), toll-like receptor signaling pathway (ssc04620), NF-κB signaling pathway (ssc04064), and the chemokine signaling pathway (ssc04062) (Figure 3B). These pathways were mainly regulated to respond to early-stage FMDV infection.

### 3.7. RT-QPCR Validation

**Figure 4:**
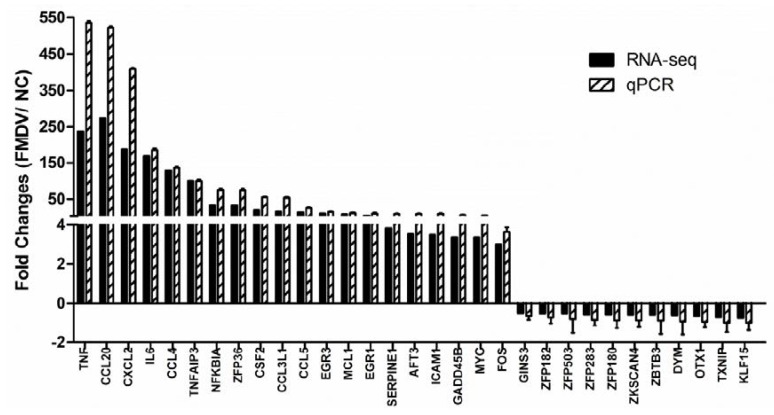
Verification of RNA-seq-detected DEGs with RT-qPCR. PK-15 cells were infected with FMDV (MOI 0.1) for 1 h and then RT-qPCR was performed to detect the relative expression of selected DEGs. The *x*-axis indicates the names of detected genes and the *y*-axis represents the fold changes after FMDV infection, β-Actin was used as an internal control.

To verify the accuracy of the RNA-seq data, RT-qPCR was performed to detect the expression change in the DEGs in PK-15 cells treated with or without FMDV for one hour. β-actin mRNA was amplified as the endogenous control. Based on the previous GO analysis of the immune-related DEGs (Table 4), 30 were selected for RT-qPCR to further investigate the expression profiles, and their primers were designed by Primer 3.0. As shown in Figure 4, the qPCR verified the results had a similar pattern to RNA-seq, with 19 up-regulated and 11 down-regulated genes. Among them, TNF(XLOC_025777) exhibited the largest up-regulated fold-change, followed by CXCL2 (XLOC_026995), CCL20 (XLOC_010559), CCL4 (XLOC_005525), ZFP36 (XLOC_009718), FOS (XLOC_025413), IL6 (XLOC_029377), NFKBIA (XLOC_025200), ICAM1 (XLOC_014586), CCL3L1 (XLOC_004911), GADD45B (XLOC_015887), VEGFA (XLOC_025051), MYC (XLOC_019591), MCL1 (XLOC_019322), EGR1 (XLOC_015003), EGR3 (XLOC_009032), CSF2 (XLOC_014957), TNFAIP3 (XLOC_001371), and CCL5 (XLOC_004915). However, thioredoxin-interacting protein (TXNIP) (XLOC_019343) displayed the largest down-regulation, followed by ZBTB3 (XLOC_014128), ZNF503 (XLOC_024457), ZNF283 (XLOC_022417), DYM (XLOC_000325), OTX1 (XLOC_018137), ZKSCAN4 (XLOC_024840), ZNF180 (XLOC_023538), ZNF182 (XLOC_032795), GINS3 (XLOC_022191), and KLF15 (XLOC_032103). To verify the results of RT-qPCR, we performed Western Blotting by using a specific NF-κB antibody (D14E12 XP Rabbit mAb, CST) against PK-15 cells. Just as Supplementary Figure S3 shown, the level of NF-κB protein was up-regulated after FMDV infection for 1 h. These cytokines and transcription factors are especially important in the immune system and play a key role in the host’s early response to FMDV infection.

## 4. Discussion

Foot-and-mouth disease (FMD) is caused by the Foot and mouth disease virus (FMDV). FMD is a highly contagious and economically devastating disease in domestic and wild cloven-hoofed animals. Upon infection, FMDV elicits a rapid and broad spectrum of immune responses, including humoral and cellular responses that induce an efficient protection against reinfection from homologous and antigenically-related viruses [19]. Neutralizing antibodies directed against well-characterized epitopes located on the viral capsid can be detected soon after infection or vaccination with FMDV. Upon early FMDV infection or vaccination, a detectable antibody response occurs in the secretions of the upper respiratory and gastrointestinal tracts [19]. The major antibody subclasses found in secretions are initially IgM, then followed by IgA and IgG [20]. The first neutralizing antibodies, IgMs, appear as early as three to four days following infection or vaccination [21].

However, little is known about the contribution of the innate immune responses in FMDV infected animals. Like many other viruses, FMDV has evolved various strategies to counteract the host immune response using several viral encoded proteins. To facilitate its replication, the virus must first overcome the host innate response. FMDV accomplishes this by inhibiting the induction of antiviral molecules at both the transcriptional and translational levels. In addition, FMDV also interferes with the secretory pathway, by inhibiting the release of IFNs and other cytokines that might adversely affect its replication and dissemination [22,23]. Studies have shown that IFN-α, β, and γ are involved in the host’s defense against FMDV infection [24]. In addition to the IFNs, other cytokines may also play a role in the host response. Conventional FMD vaccinated pigs were reported to not exhibit a systemic inflammatory response. However, the chemotactic activity of plasma on peripheral blood leukocytes increased within the first week of immunization [25]. Furthermore, in pigs that were only vaccinated or vaccinated and then challenged, the levels of interleukin-6 (IL-6), IL-8, and IL-12 in plasma increased after vaccination and/or challenge, suggesting the monocyte or macrophage was activated [26]. Although the levels of IL-6 and IL-8 did not appear to be related to the protection of pigs upon challenge, IL-12 levels were higher in vaccinated pigs, which were protected from contact challenge, suggesting a role of cytokine-induced monocytic cell activity in the protection from acute-phase disease [26].

By using RNA-seq and bioinformatics, compared with the non-treated control, in PK-15 cells that were treated with FMDV for one hour, we found that the pathways were mainly enriched in the TNF signaling pathway, cytokine-cytokine receptor interaction, NOD-like receptor signaling pathway, toll-like receptor signaling pathway, NF-κB signaling pathway, and the chemokine signaling pathway. Thirty immune-related differential genes (19 up- and 11 down-regulated) were selected for RT-qPCR verification under the criteria of a minimum two-fold change and a *p*-value less than 0.05. The results showed that the genes measured by RT-qPCR exhibited a similar expression pattern as those found with RNA-seq. Among these genes, TNF showed the largest up-regulation, followed by CXCL2, CCL20, CCL4, ZFP36, FOS, IL6, and NFKBIA. TXNIP showed the largest down-regulation, followed by ZBTB3, ZNF503, ZNF283, DYM, and OTX1.

As an essential part of the innate immune system, cytokines are involved in autocrine, paracrine, and endocrine signaling as immunomodulating agents, which are crucial for preventing viral infections [12]. Cytokines include chemokines, interferons, interleukins, lymphokines, and tumor necrosis factors. They are produced by a broad range of cells, including immune cells like macrophages, B lymphocytes, T lymphocytes and mast cells and other cells [27]. In our study, cytokines, including TNF, CXC chemokine (CXCL2), CC chemokine (CCL20, CCL4), interleukins (IL6), and NF-κB inhibitor alpha (NFKBIA), were significantly up-regulated shortly after FMDV infection. Supposedly, to fight against FMDV infection, TNF, CXCL2, CCL20, CCL4, and IL6 were up-regulated by the host to stimulate the inflammatory response.

NFKBIA inhibits NF-κB by masking the nuclear localization signals of NF-κB proteins and sequestering them in the cytoplasm in an inactive state, whereas NF-κB can negatively regulate inflammation [28]. Hence, NFKBIA may inhibit inflammation. Zinc finger proteins are the largest member of the transcription factor family, participating in biological processes, including development, differentiation, metabolism, and autophagy [29]. ZFP36, also known as Tristetraprolin (TTP), binds to AU-rich elements (AREs) in the 3′-untranslated regions (UTRs) of the mRNAs of some cytokines and promotes their degradation. Through this ability, ZFP36 limits the expression of a number of critical genes frequently overexpressed in inflammation [30]. Normal cellular FOS forms a part of the activator protein 1 (AP-1) transcription factor complex, which plays a pivotal role in cell growth, differentiation, and survival, as well as in the DNA damage response [31].

Zinc finger proteins (ZNF503, ZNF283), ZBTB3 (zinc finger and BTB domain containing 3), TXNIP (thioredoxin-interacting protein), dymeclin (DYM), and orthodenticle homeobox 1 (OTX1) were also down-regulated after FMDV infection. TXNIP is a major regulator of the cellular redox state by interacting with and inhibiting the anti-oxidative function of thioredoxin (TXN). Inhibitors of the TXN pathway are currently used to treat inflammatory diseases [32]. TXN’s function has been implicated in the activation of inflammasome and the induction of pro-inflammatory cytokine interleukin-1β [33]. Transcription factors, such as ZNF503, ZNF283, DYM, and OTX1, that control genes involved in antiviral defense may affect downstream targets at later infection stages [10]. These proteins may play crucial roles in the early immune response in pigs to FMDV infection.

## 5. Conclusions

During early FMDV infection, a series of cytokines, such as interleukins (IL6), chemokines (CXCL2, CCL20, and CCL4), and transcription factors (ZFP36, FOS, NFKBIA, ZBTB3, ZNF503, ZNF283, DYM, and OTX1) are involved in the battle between FMDV and the host through up- or down-regulation. Combined with their features and functions, we postulated that inflammation is the main means by which the host responds first to FMDV infection. The data presented here may provide an additional panel of candidate genes that are crucial for deciphering the underlying mechanisms of FMDV infection and may contribute to the prevention and control of FMD.

## Figures and Tables

**Table 1:** Results of data filtering statistics.

Sample	Number of Raw Reads	Raw Bases	Number of Clean Reads	Clean Bases	Clean Rate (%)	Q20 (%)	Q30 (%)
NC1	69,947,474	10,471,100,044	66,625,990	9,975,061,999	95.25	97.36	93.02
NC2	73,866,982	11,049,947,666	70,335,908	10,522,323,451	95.22	97.4	93.11
NC3	81,300,244	12,158,932,746	77,781,474	11,633,597,601	95.67	97.58	93.49
NC4	81,147,108	12,142,577,269	77,706,416	11,629,317,726	95.76	97.58	93.51
FMDV1	78,462,120	11,740,662,522	74,801,356	11,193,766,188	95.33	97.53	93.4
FMDV2	72,232,906	10,805,776,246	68,752,730	10,285,816,296	95.18	97.44	93.22
FMDV3	106,781,936	15,980,867,239	98,827,180	14,792,807,205	92.55	97.11	92.11
FMDV4	108,452,172	16,218,870,628	100,499,600	15,030,783,309	92.67	97.15	92.21

**Table 2:** Alignment results.

Samples	Total Reads	Mapped Reads	Uniquely Mapped Reads	Multiple Mapped Reads
NC1	66,625,990 (100.00%)	52,009,891 (78.06%)	46,518,117 (89.44%)	5,491,774 (10.56%)
NC2	70,335,908 (100.00%)	54,883,603 (78.03%)	49,189,786 (89.63%)	5,693,817 (10.37%)
NC3	77,781,474 (100.00%)	60,847,412 (78.23%)	54,541,968 (89.64%)	6,305,444 (10.36%)
NC4	77,706,416 (100.00%)	60,412,369 (77.74%)	54,154,641 (89.64%)	6,257,728 (10.36%)
FMDV1	74,801,356 (100.00%)	57,611,053 (77.02%)	51,467,860 (89.34%)	6,143,193 (10.66%)
FMDV2	68,752,730 (100.00%)	52,393,703 (76.21%)	46,872,931 (89.46%)	5,520,772 (10.54%)
FMDV3	98,827,180 (100.00%)	75,927,134 (76.83%)	67,639,000 (89.08%)	8,288,134 (10.92%)
FMDV4	100,499,600 (100.00%)	77,810,999 (77.42%)	69,128,894 (88.84%)	8,682,105 (11.16%)

**Table 3:** Assembled results.

Sample	Gene Number	Transcript Number	Exon Total Length (bp)	Average Transcript Length (bp)	Max. Transcript Length (bp)	Min. Transcript Length (bp)	N50 Length (bp, without Intron)
NC1	25,335	33,078	67,074,593	2028	24,882	156	2849
NC2	25,818	33,561	68,649,462	2046	25,217	155	2875
NC3	26,538	34,605	71,034,457	2053	25,181	140	2900
NC4	27,077	35,203	71,479,661	2030	24,449	149	2859
FMDV1	25,299	33,123	66,066,603	1995	25,261	122	2814
FMDV2	25,499	33,311	65,027,535	1952	19,333	148	2769
FMDV3	28,927	37,145	73,142,162	1969	22,369	141	2872
FMDV4	29,472	37,785	73,269,692	1939	25,126	143	2839
cuffmerge	28,141	72,673	213,510,612	2938	26,354	129	4259

**Table 4:** Gene ontology (GO) analysis of immune-related DEGs.

GO ID	Category	Gene Number	Representative Genes
GO:0002376	immune system process	39	HOXB6, EPHA2, IL6, CCL5, SOCS1, IRF1, TNFRSF4, CD83, EGR1, IL1A, ZFP36L1, BATF2, CHST3, CSF2, TNF, ADM, CEBPE, CXCL2, SOX9, LIF, ULBP1, EGR3, PHLPP1, CCR7, MAP3K14, CDC37, TNFSF15, CD70, ITPKC, NFKBIA, NR4A3, CCL4, NFIL3
GO:0006955	immune response	26	CSF2, ADM, TNF, CXCL2, IL6, CCL5, LIF, ULBP1, CCR7, MAP3K14, SOCS1, TNFRSF4, IRF1, TNFSF15, CDC37, CD70, NFKBIA, CCL4, IL1A, NR4A3, NFIL3
GO:0002682	regulation of immune system process	19	TNF, SOX9, CCL5, LIF, EGR3, CCR7, PHLPP1, SOCS1, IRF1, CDC37, CD83, ITPKC, NFKBIA, NR4A3, CHST3
GO:0002520	immune system development	16	EPHA2, TNF, CEBPE, IL6, LIF, EGR3, CCR7, PHLPP1, IRF1, CD83, EGR1, NFKBIA, ZFP36L1, BATF2
GO:0002684	positive regulation of immune system process	12	IRF1, CD83, TNF, NFKBIA, CCL5, NR4A3, LIF, EGR3, CCR
GO:0045088	regulation of innate immune response	4	NFKBIA, SOCS1, IRF1, CDC37
GO:0050776	regulation of immune response	7	NFKBIA, SOCS1, NR4A3, IRF1, CDC37, CCR7, TNF
GO:0002683	negative regulation of immune system process	5	SOX9, NFKBIA, IRF1, TNF
GO:0002253	activation of immune response	4	NFKBIA, NR4A3, IRF1, CCR7
GO:0002252	immune effector process	8	IRF1, TNF, ITPKC, NR4A3, IL6, ULBP1, CHST3
GO:0050778	positive regulation of immune response	5	NFKBIA, NR4A3, IRF1, CCR7, TNF
GO:0045087	innate immune response	7	NFKBIA, SOCS1, CCL5, CCL4, IRF1, ULBP1, CDC37
GO:0002218	activation of innate immune response	2	NFKBIA, IRF1
GO:0002697	regulation of immune effector process	4	ITPKC, NR4A3, CHST3, TNF
GO:0002366	leukocyte activation involved in immune response	3	IL6, NR4A3
GO:0045089	positive regulation of innate immune response	2	NFKBIA, IRF1
GO:0002521	leukocyte differentiation	13	EPHA2, IRF1, CD83, TNF, CEBPE, EGR1, IL6, LIF, ZFP36L1, BATF2, EGR3, CCR7
GO:0097529	myeloid leukocyte migration	6	CCL5, CCL4, CCR7
GO:0007159	leukocyte cell-cell adhesion	11	IRF1, CD83, TNF, EGR1, NR4A3, CCL5, IL6, ZFP36L1, EGR3, CCR7
GO:0045321	leukocyte activation	13	IRF1, CD83, EGR1, IL6, NR4A3, CCL5, ZFP36L1, BATF2, EGR3, CCR7
GO:0050900	leukocyte migration	7	CCL5, CCL4, CCR7, TNF
GO:0002274	myeloid leukocyte activation	5	CCL5, NR4A3, BATF2
GO:0070486	leukocyte aggregation	10	IRF1, CD83, EGR1, NR4A3, CCL5, IL6, ZFP36L1, EGR3, CCR7

## Supplementary Materials

The following are available online at http://www.mdpi.com/1999-4915/10/7/364/s1, Figure S1: Localization of FMDV at a lower magnification (40× 1.3 oil), Figure S2: Localization of FMDV at a higher magnification (100× 1.4 oil), Figure S3: Detection of NF-κB with Western Blotting; Table S1: Abbreviations, Table S2: Primers for verification of RNA-seq-detected DEGs with RT-qPCR, Table S3: Sample correlation coefficient matrix.
