# Supplementary material for: Transcript Profiling Identifies Early Response Genes against FMDV Infection in PK-15 Cells
Source: Viruses. 2018 Jul 11;10(7):364. doi: 10.3390/v10070364 (PMC6071144; doi:10.3390/v10070364)
Supplement: Supplementary file 1 [file viruses-10-00364-s001.pdf]

Supplementary Materials:

Supplemental Table 1 Abbreviations

FMDV	Foot and Mouth Disease Virus
FMD	Foot and Mouth Disease
NC	Non-treated Control
DEGs	Differentially Expressed Genes
RNA-seq	High-throughput Sequencing of Mrna
RT-qPCR	Quantitative Real-time Reverse Transcriptase PCR
$TCID_{50}$	50% Tissue Culture Infective Doses
CPE	Cytopathic Effect
MOI	Multiplicity of Infection
DMEM	Dulbecco's Modified Eagle Medium
FBS	Fetal Bovine Serum
PBS	Phosphate Buffer Saline
QC	Quality Control
FPKM	Fragments per Kilo bases per Million fragments method
GO	Gene Ontology
KEGG	Kyoto Encyclopedia of Genes and Genomes
R	Pearson Correlation Coefficient
NFKBIA	NF-kappa-B Inhibitor alpha
IL6	Interleukin 6
CCL4	C-C motif Chemokine 4
CXCL2	C-X-C motif Chemokine 2
TNF	Tumor Necrosis Factor
VEGFA	Vascular Endothelial Growth Gactor A
CCL20	C-C motif Chemokine 20
CSF2	Macrophage Colony-Stimulating Factor 2
GADD45B	Growth Arrest and DNA Damage Inducible 45 beta
MYC	Myc proto-oncogene protein
FOS	Proto-oncogene c-Fos
MCL1	Induced myeloid leukemia cell differentiation protein Mcl-1
MAP3K14	Mitogen-activated protein kinase kinase kinase 14
IRF1	Interferon regulatory factor 1
CCL5	C-C motif chemokine 5
ZBTB3	Zinc finger and BTB domain containing 3
OTX1	Orthodenticle homeobox 1
TXNIP	Thioredoxin-interacting protein
ZNF180	Znc Finger Protein 180
ZNF36	Znc Finger Protein 36
ZNF182	Zinc finger protein 182
GINS3	GINS complex subunit 3
KLF15	Kruppel-like factor 15

Supplemental Table 2 Primers for Verification of RNA-seq-detected DEGs with RT-qPCR

**TNF**	**F:** CGACTCAGTGCCGAGATCAA **R:** CTCACAGGGCAATGATCCCA
**CCL20**	**F:** AGCAACTTTGACTGCTGCCT **R:** GATCTGCACACACGGCTAAC
**CXCL2**	**F:** CCACTGTGACCAAACGGAAG **R:** ATCAGTTGGCACTGCTCTTGT
**IL6**	**F:** TGGCAGAAAAAGACGGATGC **R:** ACAGCCTCGACATTTCCCTT
**CCL4**	**F:** ATGAAGCTCTGCGTGACTGT **R:** AGTCACGAAGTTGCGAGGAA
**TNFAIP3**	**F:** TCCTCTGAAGGTGGACGGAA **R:** ACCAGGGGGACAAAGTGTTG
**NFKBIE**	**F:** GCTGTGGCCTTGTGTGTTTT **R:** TGTCCCACTCACCAGGCTAT
**ZFP36**	**F:** CCTCCCGCTACAAGACTGAG **R:** ACTTGTGGCAGAGTTCCGTC
**CSF2**	**F:** TGCCATCAAAGAAGCCCTGA **R:** GCTCCTGGGGGTCAAACATT
**CCL3L1**	**F:** TTCCTCGCAAATTCGTAGCC **R:** CATTCAGCTCCAGGTCAGAGA
**CCL5**	**F:** GAAGAAATGGGTGCGGGAGT **R:** AAGTTTGCACGAGTTCAGGC
**EGR3**	**F:** CCTATACCACCACCCCAACG **R:** CGGGTGGATCTGCTTGTCTT
**MCL1**	**F:** CTCGAGTGATGGTCCACGTT **R:** ACGGTTCGATGCAGCTTTCT
**EGR1**	**F:** AGTTTGCCAGGAGCGATGAA **R:** AGGCCACACTTTTGTCTGCT
**SERPINE1**	**F:** AACCAGGCGGACTTCTCAAG **R:** TGCGGGCTGAGACGATAATG
**ATF3**	**F:** CTCTCGAATCCCAGCAGCAA **R:** CCCAGGTCCAAAGTCCACTC
**ICAM4**	**F:** GCCAGGATTACCGCCTACAA **R:** AGAGTCACCACAAGGAAGCC
**GADD45B**	**F:** GTGTCAGGAATGCAGCGACT **R:** GCATCTGTGTGAGGGTTCGT
**FOS**	**F:** GAGATGTCTGTGGCTTCCCT **R:** TCCATGCTGCTGACGTTCTT
**GINS3**	**F:** AACAAGCGGCGGATCCTTTC **R:** GAGCCGAACCCGTAGAAGTG
**ZNF182**	**F:** AGTTGTCCCAAAGCGGAAGT **R:** AGCCCATCACTCCTACCACT
**ZNF503**	**F:** ATGTTAGCTCCAAGCGGACC **R:** ATCTACAAGGGACGGGAGGG
**ZNF283**	**F:** TCTGAGGATTTTCCGGCTCT **R:** TTGAAGACACGGGAAAGGGAC
**ZNF180**	**F:** CAGCCAGAAGCGTAGCCATT **R:** GCACACGACAGACTAGGGTT
**ZKSCAN4**	**F:** TCCTTTACCTCTGGTGTGTGG **R:** GCAAACGGTCAACAAAGCCAT
**ZBTB3**	**F:** TTTCCCACGGACAAGGACAC **R:** CTTGCCCCCTTAACTAGCCC
**DYM**	**F:** GAGCAGCCCGAGGAGTTTTT **R:** CTGCGGGTTCCAGTATAGGC
**OTX1**	**F:** TTCCGTCGTCGTTGAGTACG **R:** GCATACACGATGCGTTGCTT
**TXNIP**	**F:** CATTGTGGTGCCCAAAGCTG **R:** GCCTCTGACCGATGACAACT
**KLF15**	**F:** CGACCCTGATGATGTCCCAA **R:** TTGCTGTGGCTTTCTGGGG

Supplemental Table 3 Sample correlation coefficient matrix

	**NC1**	**NC2**	**NC3**	**NC4**	**FMDV1**	**FMDV2**	**FMDV3**	**FMDV4**
NC1	1	0.981689339	0.978415833	0.97949195	0.962341549	0.960497019	0.962599115	0.95656192
NC2	0.981689339	1	0.980418147	0.980903622	0.965636153	0.964933104	0.967204742	0.961261951
NC3	0.978415833	0.980418147	1	0.981750033	0.963524847	0.961381466	0.963298739	0.95719982
NC4	0.97949195	0.980903622	0.981750033	1	0.96724223	0.96557678	0.964897729	0.960085685
FMDV1	0.962341549	0.965636153	0.963524847	0.96724223	1	0.985139979	0.980657688	0.979882408
FMDV2	0.960497019	0.964933104	0.961381466	0.96557678	0.985139979	1	0.98210694	0.980679624
FMDV3	0.962599115	0.967204742	0.963298739	0.964897729	0.980657688	0.98210694	1	0.981273647
FMDV4	0.95656192	0.961261951	0.95719982	0.960085685	0.979882408	0.980679624	0.981273647	1

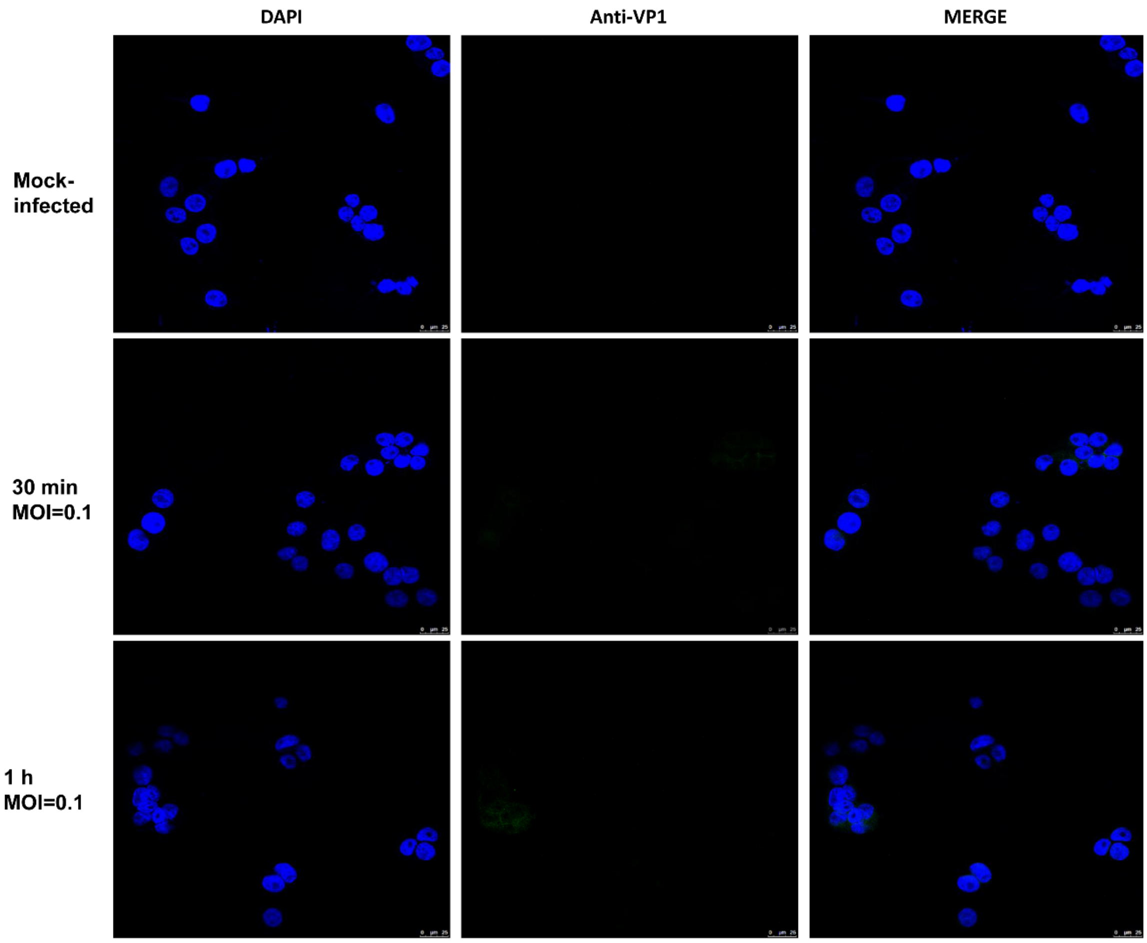

Figure S1. Localization of FMDV at a lower magnification (40× 1.3 oil). PK-15 cells were infected with FMDV (MOI 0.1) and confocal microscopy (with a 40 × 1.3 oil numerical aperture objective) was performed to detect the localization of FMDV. Anti-VP1 antibody was used to detect the capsid protein VP1, DAPI was used to show the nucleus.

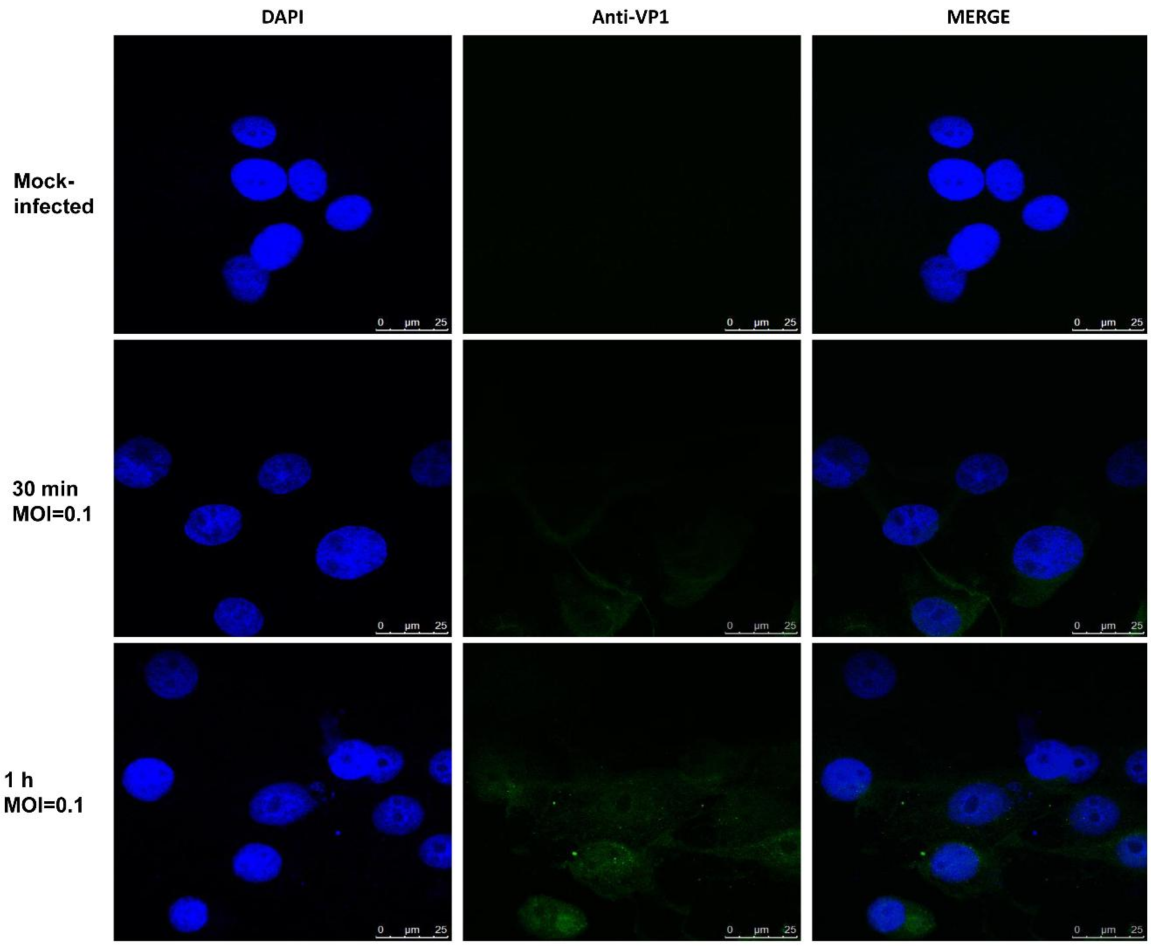

Figure S2. Localization of FMDV at a higher magnification (100× 1.4 oil). PK-15 cells were infected with FMDV (MOI 0.1) and confocal microscopy (with a 100 × 1.4 oil numerical aperture objective) was performed to detect the localization of FMDV. Anti-VP1 antibody was used to detect the capsid protein VP1, DAPI was used to show the nucleus.

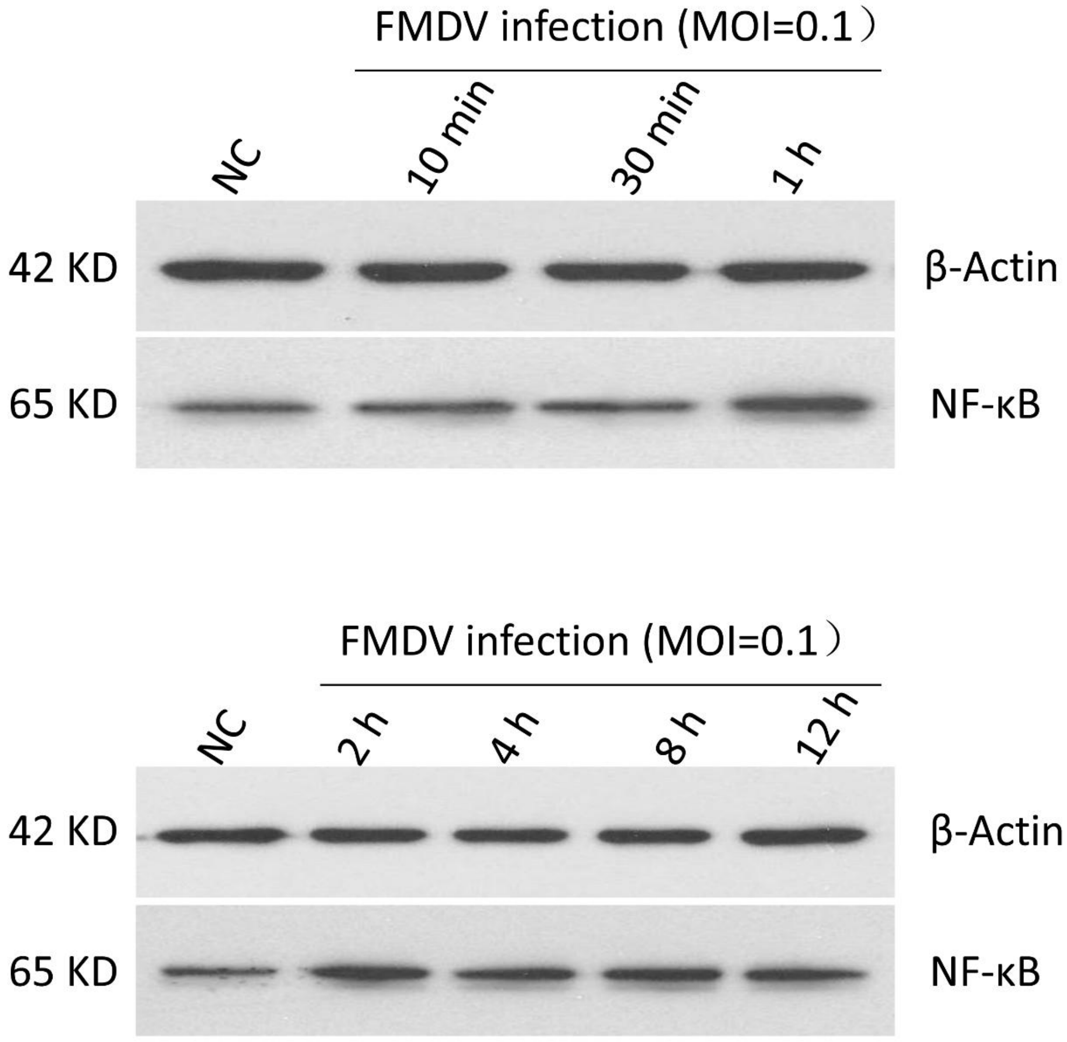

Figure S3. Detection of NF-κB with Western Blotting. PK-15 cells were infected with FMDV (MOI 0.1) and Western Blotting was performed to detect NF-κB expression from 10 min to 12 hours after infection, β-Actin was used as a loading control to show the evening loading of the samples.
